# Progression of Cardiovascular Manifestations in Adults and Children With Mucopolysaccharidoses With and Without Enzyme Replacement Therapy

**DOI:** 10.3389/fcvm.2021.801147

**Published:** 2022-01-12

**Authors:** Fabiano de Oliveira Poswar, Hallana Souza Santos, Angela Barreto Santiago Santos, Solano Vinicius Berger, Carolina Fischinger Moura de Souza, Roberto Giugliani, Guilherme Baldo

**Affiliations:** ^1^Postgraduate Program in Genetics and Molecular Biology, Federal University of Rio Grande do Sul, Porto Alegre, Brazil; ^2^Medical Genetics Service, Hospital de Clínicas de Porto Alegre, Porto Alegre, Brazil; ^3^Cardiology Service, Hospital de Clínicas de Porto Alegre, Porto Alegre, Brazil; ^4^Postgraduate Program in Cardiology and Cardiovascular Sciences, Medical School, Federal University of Rio Grande do Sul, Porto Alegre, Brazil; ^5^Department of Genetics, Federal University of Rio Grande do Sul, Porto Alegre, Brazil; ^6^Postgraduate Program in Physiology, Federal University of Rio Grande do Sul, Porto Alegre, Brazil

**Keywords:** mucopolysaccharidoses, enzyme replacement therapy, pulmonary hypertension, left ventricular hypertrophy, left atrium, heart valve disease

## Abstract

**Background:** Cardiovascular involvement is among the main features of MPS disorders and it is also a significant cause of morbidity and mortality. The range of manifestations includes cardiac valve disease, conduction abnormalities, left ventricular hypertrophy, and coronary artery disease. Here, we assessed the cardiovascular manifestations in a cohort of children and adults with MPS I, II, IV, and VI, as well as the impact of enzyme replacement therapy (ERT) on those manifestations.

**Methods:** We performed a chart review of 53 children and 23 adults with different types of MPS that had performed echocardiograms from January 2000 until October 2018. Standardized Z scores were obtained for heart chamber sizes according to the body surface area. When available, echocardiographic measurements that were performed before ERT and at least 18 months after that date were used for the assessment of pre- and post-treatment parameters.

**Results:** Left side valvular disease was a frequent finding, with mitral and aortic thickening being reported in most patients in all four MPS types. Left atrium dilatation was present in 26% of the patients; 25% had increased relative wall thickness; 28% had pulmonary hypertension. The cardiovascular involvement was, in general, more prevalent and more severe in adults than in children, including conduction disorders (40 vs. 16%), mitral stenosis (26 vs. 6%), aortic stenosis (13 vs. 4%), and systolic dysfunction (observed in only one adult patient). ERT promoted a significant reduction of the left ventricular hypertrophy parameters, but failed to improve valve abnormalities, pulmonary hypertension, and left atrial dilatation.

**Conclusions:** Adult patients with MPS may develop severe cardiovascular involvement, not commonly observed in children, and clinicians should be aware of the need for careful monitoring and timely management of those potentially life-threatening complications. Our results also confirm the impact of long-term ERT on left ventricular hypertrophy and its limitations in reversing other prevalent cardiovascular manifestations.

## Introduction

The mucopolysaccharidoses (MPS) are a group of eleven disorders characterized by impaired catabolism of glycosaminoglycans (GAGs) as a consequence of a deficiency of lysosomal enzymes directly involved in their degradation, resulting in the accumulation of one or more of five different types of GAGs: heparan sulfate, dermatan sulfate, keratan sulfate, chondroitin sulfate, and hyaluronan (1). According to the accumulated substrate and clinical features, the MPS disorders are classified into seven main types (I, II, III, IV, VI, VII, and IX), with MPS III and MPS IV being further subclassified in four (IIIA, IIIB, IIIC, and IIID) and two (IVA and IVB) subtypes, respectively, according to the enzymatic defect (2).

Cardiovascular involvement is among the main features of MPS disorders and it is also a significant cause of morbidity and mortality. As GAGs are a significant normal component of cardiac structures, enzymatic deficiencies related to GAG degradation result in prominent storage of undegraded GAGs in heart structures, which may cause tissue damage through the activation of cell proteases (3, 4). This results in many different manifestations, including cardiac valve disease, conduction abnormalities, hypertrophy of the left ventricle, and coronary artery disease (3).

Replacing the deficient enzyme, either through Enzyme Replacement Therapy (ERT) or Hematopoietic Stem Cell Transplantation (HSCT), is the current paradigm of targeting the primary defect in MPS. HSCT is widely used in the severe form—Hurler phenotype—of MPS I and ERT is available for the treatment of MPS types I, II, IVA, VI, and VII (5). Nevertheless, real-world experience with those disease-modifying therapies has unveiled several limitations, especially regarding their ability to reverse or even stop the progression of some cardiovascular manifestations in MPS patients (5, 6). In recent years, as the management of patients of MPS is resulting in an increased life expectancy, those progressing complications are a growing concern for healthcare providers (7).

In this study, we aimed to assess the cardiovascular manifestations in children and adults with different MPS types, as well as the impact of enzyme replacement therapy on those manifestations.

## Methods

### Data Collection

Following institutional ethical approval (17-0013; Grupo de Pesquisa e Pós Graduação, Hospital de Clínicas de Porto Alegre, Porto Alegre, Brazil), we performed a chart review of patients with MPS that had performed echocardiograms from January 2000 until October 2018. When available, electrocardiographic data were also recorded. MPS III patients were excluded from the study because there is an insufficient number of individuals in our center with available echocardiograms or electrocardiograms results.

Body surface area was calculated using the geometric method of Haycock (8). Left ventricular mass (LVM) was obtained using the formula of Devereaux (9). Standardized Z scores for left ventricle posterior wall thickness (LVPWT) and interventricular septum thickness (IVST) were calculated using the methods described by Lopez et al. (10), while, for the left ventricular mass (LVM) and left atrium parasternal long axis anteroposterior dimension (LAD) an online resource from Boston Children's Hospital Heart Center was used (11, 12). Relative wall thickness was obtained using the formula (2 × LVPWT)/(LV internal diameter at end-diastole) and a cut-off value of 0.41 was used for classification of the left ventricle geometry, as previously described (13). Left ventricle ejection fraction (LVEF) was obtained through the method of Teichholz (14) and systolic pulmonary artery pressure was obtained with doppler flow studies. For the analysis of the prevalence of the cardiac manifestations, the last available echocardiogram was used. When available, echocardiographic measurements performed before ERT (up to 18 months before ERT was started) and at least 18 months after that date (up to 166 months after ERT was started) were used for the assessment of pre- and post-treatment measurements. Those patients who received ERT in the peri-transplant period or as a concomitant therapy after transplantation were not included in that assessment. Changes in echocardiographic measurements were also analyzed for treatment naïve patients that had at least two echocardiograms performed with a minimum interval of 18 months.

For those who also had an available electrocardiogram, the presence or absence of left atrial enlargement, repolarization disorder, and left ventricular hypertrophy according to the clinical report were recorded. Interval measurements and Z scores for heart rate vs. age were also obtained (11, 12). Corrected QT (QTc) interval was calculated using the Bazett formula (15).

### Statistical Analysis

All data were entered into PASW Statistics 18.0 for Windows (SPSS Inc., Chicago, IL, USA) and submitted to specific statistical analysis. Graphics and part of the statistic tests were generated using the GraphPad Prism version 7.0. The normality of the samples was assessed with Shapiro-Wilk and D'Agostino and Pearson tests. Non-parametric Kruskal-Wallis and Dunn's post-hoc tests were used for the comparison of quantitative measurements among different MPS types. Mann-Whitney U test was used for comparisons of quantitative measurements between adults and children. A chi-squared test was used for comparison of the frequencies of abnormalities among MPS types and between children and adults. For the comparison between baseline and follow-up parameters after ERT, the parametric paired t-test was used to assess Z-scores of cardiac structures and the Wilcoxon matched-pairs signed-rank test was used to assess cardiac valves. A p-value of <0.05 was considered significant.

## Results

A total of 76 patients (27 MPS I, 22 MPS II, 19 MPS IVA, and 8 MPS VI), 46 males and 30 females, with a mean age of 14.2 years, being 53 children and 23 adults, were included in this study (Tables 1, 2). Most of those patients were analyzed in a previous publication, which focused on the aortic root dimension (16). All MPS II patients were males. MPS IVA patients had a lower median height, when compared to MPS II patients and were less frequently treated with ERT when compared to MPS VI patients.

**Table 1:** Characteristics of the subjects according to MPS type.

	**Overall (*n* = 76)**	**MPS I (*n* = 27)**	**MPS II (*n* = 22)**	**MPS IVA (*n* = 19)**	**MPS VI (*n* = 8)**	***p*-value**
Sex (*n*)	M: 46 F: 30	M: 12 F: 15	M: 22 F: 0	M: 8 F: 11	M: 4 F: 4	<0.001†
Age (years)	11.3 (12.7)	10.2 (20.4)	11.8 (15.5)	10.1 (8.5)	16.2 (8.3)	0.491
Number of adults (*n*)	23/76 (30%)	8/27 (29%)	5/22 (23%)	7/19 (37%)	3/8 (38%)	0.757
Weight (kg)	25.6 (21.0)	23.5 (23.4)	28.0 (36.5)	21.0 (16.6)	22.3 (14.9)	0.103
Height (cm)	111.0 (35.0)	108.0 (8.4)	129.0 (36.0)	99.5 (19.7)	106.5 (18.4)	0.003^‡^
BSA (m^2^)	0.9 (0.5)	0.9 (0.6)	1.0 (0.7)	0.8 (0.3)	0.8 (0.4)	0.057
Treated with ERT (*n*)	47/76	16/27^*^	15/22	8/19	8/8	0.029^§^
Age at the start of ERT (years)	8.3 (10.1)	4.0 (15.4)	6.6 (8.9)	8.3 (8.1)	9.7 (2.3)	0.538
Time on ERT (months)	41.0 (77.0)	52.0 (79.5)	64.0 (76.0)	22.5 (22.3)	73.5 (79.0)	0.317

*Continuous variables are reported as median and interquartile range*.

* *Four patients who received ERT as a concomitant therapy for HSCT are not included in the ERT-treated group. Statistical analysis with chi-squared and partitioning for categorical variables and Kruskal-Wallis test with Dunn's post-hoc test for continuous variables*.

† *MPS II is different from other MPS types*.

‡ *MPS IVA is different from MPS II*.

§ *MPS IVA is different from MPS VI*.

**Table 2:** Characteristics of the subjects according to the age group.

	**Overall (*n* = 76)**	**Children (*n* = 53)**	**Adults (*n* = 23)**	***p*-value**
Sex (*n*)	M: 46 F: 30	M: 34 F: 19	M: 12 F: 11	0.444
Age (years)	11.3 (12.7)	9.2 (7.3)	26.7 (9.8)	<0.001^†^
Weight (kg)	25.6 (21.0)	21.5 (10.0)	40.0 (32.0)	<0.001^†^
Height (cm)	111.0 (35.0)	104.0 (22.0)	128.5 (42.0)	<0.001^†^
BSA (m^2^)	0.9 (0.5)	0.80 (0.29)	1.21 (0.62)	<0.001^†^
Treated with ERT (*n*)	47/76^*^	32/53^*^	15/23	0.799
Age at the start of ERT (years)	8.3 (10.1)	6.3 (6)	17.1 (6.25)	<0.001^†^
Time on ERT (months)	41.0 (77.0)	33.5 (58)	107.06 (65)	<0.001^†^

*Continuous variables are reported as median and interquartile range*.

* *Four children who received ERT as a concomitant therapy for HSCT are not included in the ERT-treated group. Statistical analysis with chi-squared for categorical variables and Mann-Whitney U test for continuous variables*.

† *p < 0.05*.

### Valvular Disease

**Figure 1:**
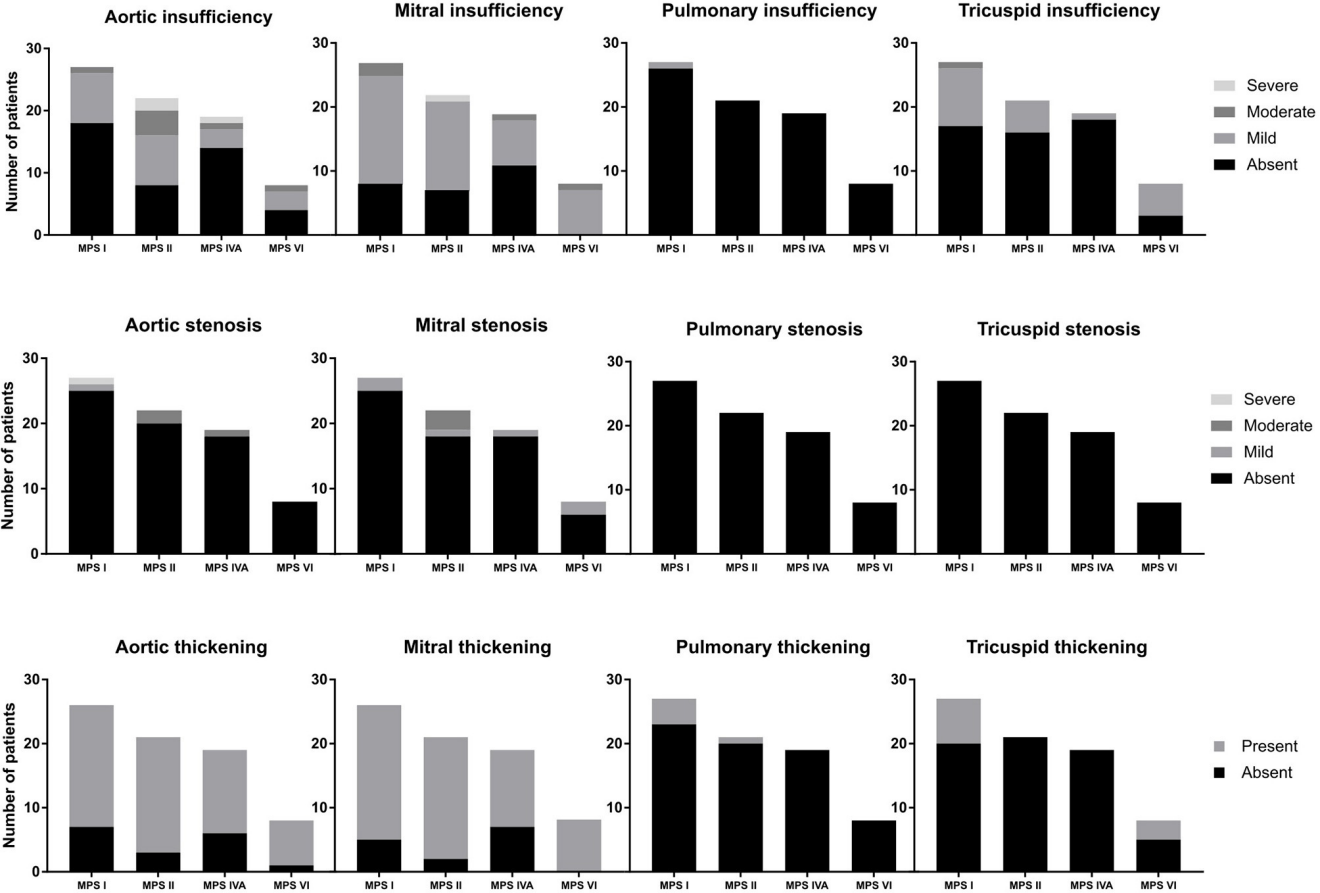
Prevalence of heart valve abnormalities in MPS types I, II, IVA, and VI, including both treated and untreated subjects.

**Figure 2:**
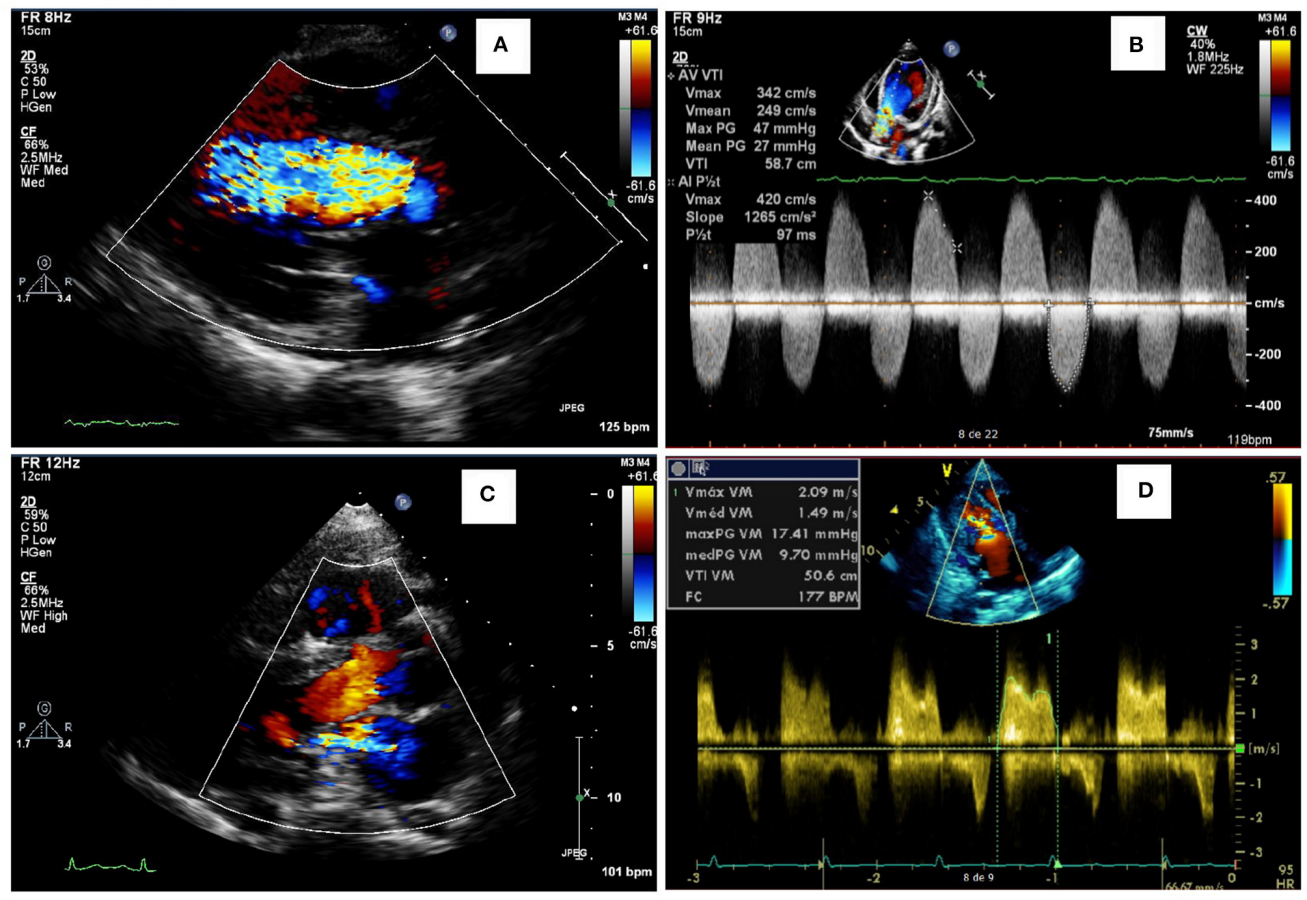
Representative abnormalities identified in the valves of the patients of this study. Aortic insufficiency **(A)** and aortic stenosis **(B)** in a female in the age range of 26–30 years with MPS IVA, who also had a reduced ejection fraction (35%). Mitral insufficiency **(C)** in a male in the age range of 21–25 years with MPS VI. Mitral stenosis **(D)** in an adolescent male with MPS II.

Among the 76 patients included in this study, left side valvular disease was a frequent finding, with mitral and aortic thickening being reported in most patients in all four MPS types (Figure 1). Furthermore, mitral and aortic insufficiency, mostly mild, were frequently found in patients with MPS I, II, and VI, but were also observed in a significant proportion of patients with MPS IVA. To a lesser extent, tricuspid valve thickening and insufficiency were also present in patients with MPS types I, II, and VI. Heart valve involvement was both more prevalent and more severe in adult patients (Figure 2; Table 3).

**Table 3:** Prevalence of echocardiographic abnormalities in children and adults with MPS.

	**Overall (*n* = 76)**	**Children (*n* = 53)**	**Adults (*n* = 23)**	***p*-value**
Aortic insufficiency	32/76 (42%)	17/53 (32%)	15/23 (65%)	0.011^*^
Aortic stenosis	5/76 (7%)	2/53 (4%)	3/23 (13%)	0.159
Pulmonary insufficiency	0/76 (0%)	0/53 (0%)	1/23 (4%)	0.303
Pulmonary stenosis	0/76 (0%)	0/53 (0%)	0/23 (0%)	n/a
Mitral insufficiency	50/76 (66%)	31/53 (59%)	19/23 (83%)	0.064
Mitral stenosis	9/76 (12%)	3/53 (6%)	6/23 (26%)	0.019^*^
Tricuspid insufficiency	21/76 (28%)	16/53 (30%)	5/23 (22%)	0.580
Tricuspid stenosis	0/76 (0%)	0/53 (0%)	0/23 (0%)	n/a
LVM Z score > 2	6/76 (8%)	5/53 (9%)	1/23 (4%)	0.661
RWT > 0.41	19/76 (25%)	15/53 (28%)	4/23 (17%)	0.395
Concentric remodeling	16/76 (21%)	12/53 (16%)	4/23 (17%)	0.622
Concentric hypertrophy	3/76 (4%)	3/53 (6%)	0/23 (0%)	
Eccentric hypertrophy	3/76 (4%)	2/53 (4%)	1/23 (5%)	
LAD Z score > 2	20/76 (26%)	11/53 (21%)	9/23 (39%)	0.155
SPAP > 35 mmHg^*^	11/40 (28%)	8/28 (29%)	3/12 (25%)	1.000
LVEF <55%	1/76 (1%)	0/53 (0%)	1/23 (5%)	0.303

*LAD, Left atrial diameter; LVEF, Left ventricle ejection fraction; LVM, left ventricle mass; RWT, relative wall thickness; SPAP, systolic pulmonary artery pressure. Statistical analysis with Fisher's exact test*.

* *p < 0.05*.

### Other Echocardiographic Parameters

**Figure 3:**
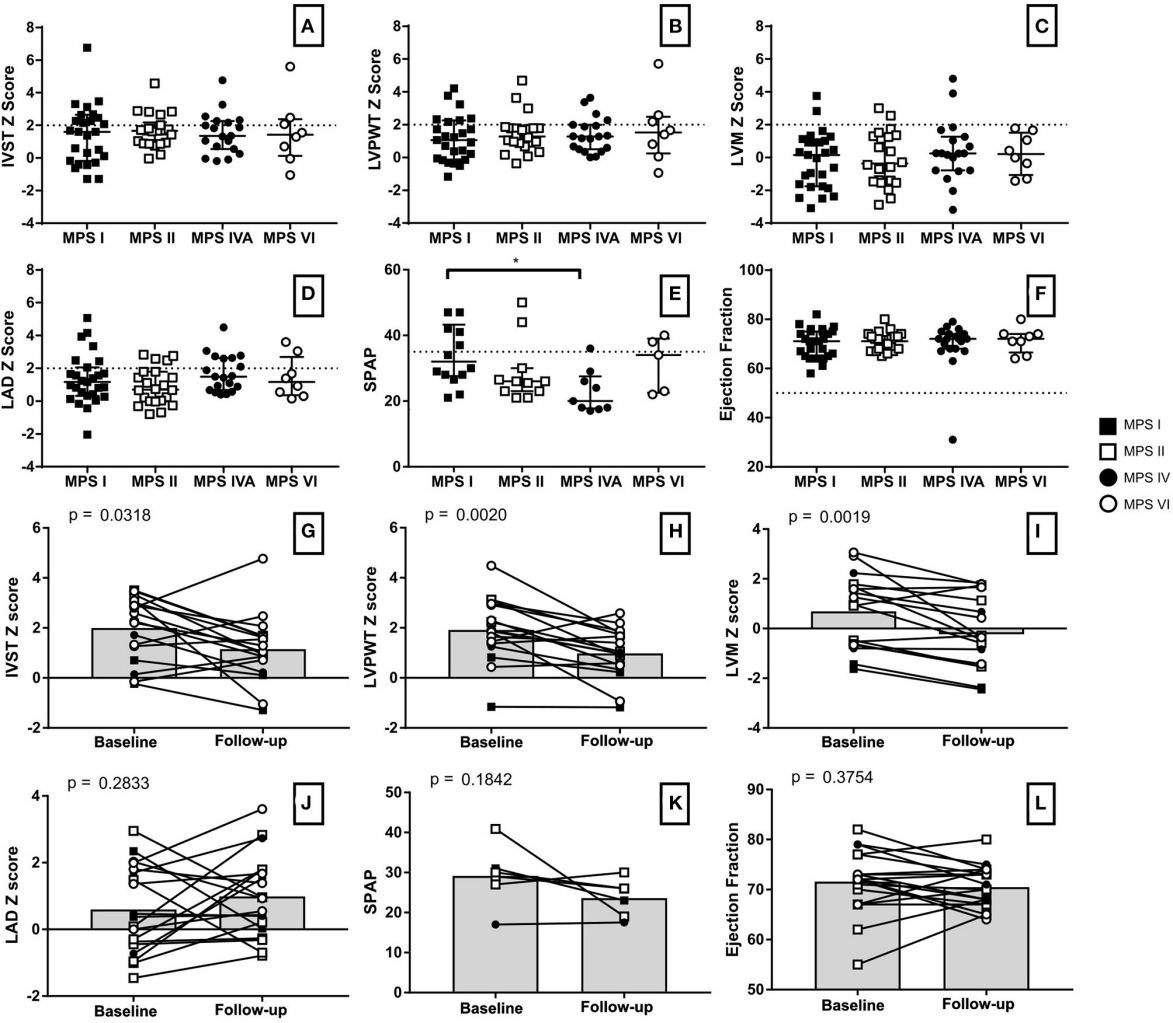
Measurements of echocardiographic parameters among MPS types **(A–F)** as well as before and after ERT for single individuals **(G–L)**, including the Z scores of interventricular septum thickness **(A,G)**, left ventricle posterior wall thickness **(B,H)**, left ventricle mass **(C,I)**, left atrium diameter **(D,J)**, systolic pulmonary artery pressure **(E,K)** and ejection fraction **(F,L)**. A school-aged girl with severe MPS I fell outside the axis of both IVST **(A)** and LVPWT, with Z scores values of 9.0 and 9.7, respectively. Another MPS I patient (a female in the age range of 26–30 years with Hurler-Scheie phenotype) fell outside the axis range of SPAP **(E)**, because she had an SPAP of 110 mmHg. Statistical analysis with Kruskal-Wallis and Dunn's post-hoc test for comparisons among MPS types **(A–F)** and with paired t-test for before and after analyses **(G–L)**. *p < 0.05. MPS IVA is different from MPS I.

Left ventricular hypertrophy parameters, including LVPWT, IVST Z scores, and RWT, were above average in a significant proportion of patients in all subgroups (Figure 3). Nevertheless, LVM Z scores were normal in the last available echocardiogram of most of the patients (Table 2). Signs of left ventricular hypertrophy were more commonly observed in children than in adults (Table 3).

When assessed the last available echocardiogram, the median left atrium diameter (LAD) and the estimated systolic pulmonary artery pressure (SPAP) were increased in patients with all types of MPS (Figures 3D,E). A total of 26 and 28% of the patients had LAD or SPAP above normal reference limits, respectively (Table 4). The median value of SPAP was significantly lower in MPS IVA than in MPS I (Figure 3D). Left ventricular ejection fraction was preserved in most patients, except for one adult patient with MPS IVA, who also had eccentric hypertrophy, severe aortic insufficiency, and moderate aortic stenosis (Figures 2A,B, 3F).

**Table 4:** Prevalence of echocardiographic abnormalities at the last available echocardiogram, including both ERT treated and untreated subjects.

	**Overall (n = 76)**	**MPS I (n = 27)**	**MPS II (n = 22)**	**MPS IV (n = 19)**	**MPS VI (n = 8)**	**p-value**
LVM Z score > 2	6/76 (8%)	2/27 (7%)	2/22(9%)	2/19 (11%)	0/8 (0%)	0.821
RWT > 0.41	19/76 (25%)	7/27 (26%)	7/22 (32%)	3/19 (16%)	2/8 (25%)	0.701
Concentric remodeling	16/76 (21%)	5/27 (19%)	6/22 (27%)	3/19 (16%)	2/8 (25%)	0.697
Concentric hypertrophy	3/76 (4%)	0/27 (0%)	1/22 (5%)	2/19 (11%)	0/8 (0%)	
Eccentric hypertrophy	3/76 (4%)	2/27 (7%)	1/22 (5%)	0/19 (0%)	0/8 (0%)	
LAD Z score > 2	20/76 (26%)	7/27 (26%)	4/22 (18%)	7/19 (37%)	2/8 (25%)	0.605
SPAP > 35 mmHg^*^	11/40 (28%)	6/14 (43%)	2/12 (17%)	1/9 (11%)	2/5 (40%)	0.265
LVEF <55%	1/76 (1%)	0/27 (0%)	0/22 (0%)	1/19 (5%)	0/8 (0%)	0.385

*LAD, Left atrial diameter; LVEF, Left ventricle ejection fraction; LVM, left ventricle mass; RWT, relative wall thickness; SPAP, systolic pulmonary artery pressure*.

* *SPAP was measured in 40 of the total 76 available last echocardiograms of the participants. Statistical analysis with chi-squared*.

### Electrocardiogram

**Figure 4:**
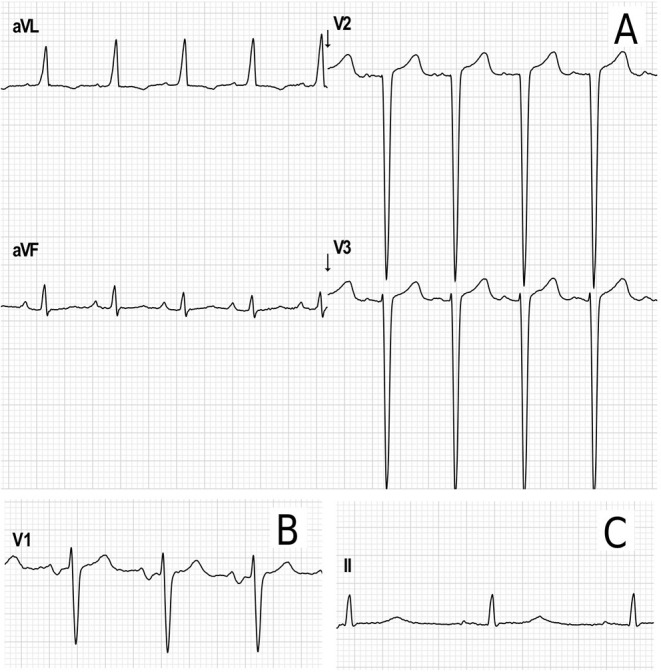
Representative electrocardiographic abnormalities identified in the adult patients of this study. **(A)** Electrocardiographic criteria for left ventricular hypertrophy (Cornell index: 40 mm) and repolarization abnormalities in a female in the age range of 26–30 years with MPS IVA. **(B)** Left atrium enlargement in a male in the age range of 21–25 years with MPS II (terminal negative deflection within the P wave in lead V1). **(C)** Increased PR interval (203 ms) and transient prolonged QTc (464 ms) in female in the age range of 31–35 years with MPS I during postoperative care for aortic valve and mitral valve replacement.

Electrocardiograms were performed in 65 patients (45 children and 20 adults) at some point during follow-up. All patients had a sinus rhythm, although transient junctional rhythm was observed for one female with MPS IVA in the middle childhood. The most frequently observed finding was the presence of repolarization anomalies (29%) (Table 5; Figure 4A). Left ventricular hypertrophy (LVH) and left atrium enlargement were present in 5 and 8% of the patients, respectively. Atrioventricular block and long QT intervals were also occasionally observed (Figure 4C), as well as intraventricular blocks. As compared to children, adults had a higher prevalence of most of the ECG abnormalities (Table 6), particularly ventricular repolarization abnormalities (Figure 4A) and left atrial enlargement (Figure 4B).

**Table 5:** Prevalence of electrocardiographic abnormalities at the last available electrocardiogram, including both ERT treated and untreated subjects.

	**Overall (*n* = 65)**	**MPS I (*n* = 20)**	**MPS II (*n* = 19)**	**MPS IV (*n* = 19)**	**MPS VI (*n* = 7)**	***p*-value**
Ventricular repolarization abnormalities	19/65 (29%)	4/20 (20%)	6/18 (33%)	8/19 (42%)	1/8 (13%)	0.311
Left atrial enlargement	5/65 (8%)	2/20 (10%)	2/18 (11%)	0/19 (0%)	1/8 (13%)	0.514
Right axis deviation	5/65 (8%)	2/20 (10%)	0/18 (0%)	1/19 (5%)	2/8 (25%)	0.159
Left ventricular overload	3/65 (5%)	1/20 (5%)	1/18 (5%)	1/19 (5%)	0/8 (0%)	0.930
Right ventricular overload	3/65 (5%)	0/20 (0%)	2/18 (11%)	1/19 (5%)	0/8 (0%)	0.377
Intraventricular block	3/65 (5%)	1/20 (5%)	0/18 (0%)	0/19 (0%)	2/8 (25%)	0.025^*^
Atrioventricular block	3/65 (5%)	2/20 (10%)	1/18 (5%)	0/19 (0%)	0/8 (0%)	0.447
Right atrial enlargement	2/65 (3%)	2/20 (10%)	0/18 (0%)	0/19 (0%)	0/8 (0%)	0.200
Prolonged QRS	1/65 (2%)	0/20 (0%)	1/18 (5%)	0/19 (0%)	0/8 (0%)	0.448

*Statistical analysis with chi-squared*.

* *p < 0.05*.

**Table 6:** Prevalence of abnormalities in the resting electrocardiogram in children and adults with MPS.

	**Overall (*n* = 65)**	**Children (*n* = 45)**	**Adults (*n* = 20)**	***p*-value**
Ventricular repolarization abnormalities	19/65 (29%)	7/45 (16%)	12/20 (60%)	0.001^*^
Left atrial enlargement	5/65 (8%)	0/45 (0%)	5/20 (25%)	0.002^*^
Right axis deviation	5/65 (8%)	2/45 (4%)	3/20 (15%)	0.165
Left ventricular overload	3/65 (5%)	2/45 (4%)	1/20 (5%)	1.000
Right ventricular overload	3/65 (5%)	3/45 (7%)	0/20 (0%)	0.547
Intraventricular block	3/65 (5%)	1/45 (2%)	2/20 (10%)	0.222
Atrioventricular block	3/65 (5%)	2/45 (4%)	1/20 (5%)	1.000
Right atrial enlargement	2/65 (3%)	2/45 (4%)	0/20 (0%)	1.000
Prolonged QRS	1/65 (2%)	1/45 (2%)	0/20 (0%)	1.000

*Statistical analysis with Fisher's exact test*.

* *p < 0.05*.

### Effects of the Enzyme Replacement Therapy

**Figure 5:**
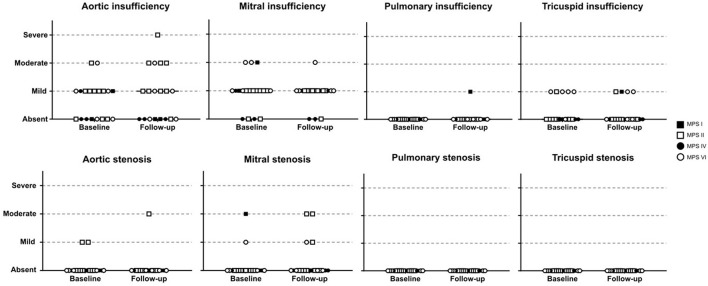
Comparison of valve abnormalities before and after ERT. In the statistical analysis, with Wilcoxon matched-pairs signed rank test, no significant difference was identified.

For those 19 patients whose echocardiographic measurements were available before and after ERT start (11 children and 8 adults), a significant reduction of LVH parameters (including IVST, LVPWT, and LVM) was observed after ERT was started (Figures 3G–I), a finding that was not identified in 18 patients that remained untreated with ERT (Supplementary Figure 1). However, no statistically significant changes in SPAP, LAD, or LVEF were observed after ERT (Figures 3J–L). Moreover, ERT did not lead to significant improvements in valvular disease (Figure 5).

As only 4 patients had available electrocardiograms in an adequate time frame before and after the start of ERT, we could not explore the impact of ERT in conduction anomalies.

## Discussion

In this study, we assessed the prevalence of cardiac manifestations in a cohort of patients with MPS I, II, IVA, and VI; which included mostly patients treated with ERT. In agreement with previous reports (3, 17–20), valve involvement comprised mainly left-sided valves and affected a high proportion of patients. Mitral valve involvement was more common than the aortic valve in the four MPS types. Valve involvement, especially aortic insufficiency and mitral stenosis, was more commonly observed in older patients. We could not identify any significant worsening or improvement of valve pathology after ERT. It is widely accepted that ERT has limited impact on valve abnormalities of patients with MPS (13, 21–25), probably due to poor tissue penetration and irreversibility of the valvar damage. Nevertheless, it may have a role in preventing or delaying its appearance when treatment is started very early, as suggested by studies with sibling pairs and animal models (26, 27).

The finding of normal Z scores for LVM in a significant proportion of the patients in our cohort may reflect the effects of long-term therapy in this parameter. Accordingly, significant reductions of IVST, LVPWT, and LVM were observed in the follow-up measurements, which is in agreement to prior studies and generally acknowledged as a well-established effect of ERT (3, 5, 13, 22–24).

Hypertrophic cardiomyopathy (HCM) has been described in different inherited metabolic disorders and it is relatively milder in MPS as compared to other conditions such as infantile Pompe Disease or Danon disease (13, 28, 29). In patients with MPS, an increase in echocardiographic LVH parameters may reflect storage of undegraded substrate, without actual increase in muscle mass (pseudohypertrophy) and/or the activation of complex pathways triggered by the GAG deposition, causing HCM (28–30). Moreover, LVH in MPS may also be secondary to an increased workload associated with valve disease or arterial hypertension (3, 31).

In both humans and murine models of MPS, it was observed that GAG deposits occur mainly in histiocytes, while there is scant evidence of deposit in cardiomyocytes (32, 33). Furthermore, in MPS mice treated with bone marrow transplantation, no abnormal storage is detected in the heart tissue (32). Effects of ERT in left ventricular (pseudo-) hypertrophy are also likely to be secondary to its ability to clear GAG storage in the heart wall, as there is no evidence of improvement of valve disease or hypertension with the treatment.

We found a high proportion of patients with left atrial diameter Z scores above normal limits (26%), but a lower prevalence of left atrial enlargement criteria on electrocardiogram (8%). This difference may be attributed to a lower sensitivity of the latter method as an indicator of left atrial enlargement (34). Previous studies have reported the presence of left atrial dilatation in 6.3% of patients with MPS IVA (35) and 2.3% of patients with MPS VI (13). Another study reported 10.7% of MPS patients with biatrial enlargement criteria on electrocardiogram (25). As there is a higher proportion of aortic root dilatation in patients with MPS (6, 36), the use of left atrium to aortic root ratio may underestimate the true prevalence of atrial enlargement (37). Left atrial enlargement was more frequently identified in adults, and the impact of ERT in the parameter has not been established.

Systolic pulmonary artery pressure (SPAP) was increased in 28% of the patients. Other authors have reported a prevalence of 36% of pulmonary hypertension (PH) in a sample of 28 pediatric patients and emphasized that it was the main cause of death in their cohort (17). Chronic hypoxemia secondary to obstructive sleep apnea is a known cause of pulmonary hypertension among MPS patients, but it cannot explain all cases, and a role for the left heart dysfunction in its pathogenesis is also likely (17, 38, 39).

When analyzing the whole sample, we could not demonstrate a statistically significant improvement or worsening of the left ventricle ejection fraction. Nevertheless, ejection fraction was already preserved in most patients. Also, the adult MPS IVA patient reported with low LVEF in Figure 3F has never received ERT. In the literature, the impact of ERT in ejection fraction is less clear, with some studies pointing to stabilization or reduction of the mean value over time (13, 40) and others showing improvements in those patients who already had reduced LVEF on baseline (41). Although those inconsistencies need further clarification, it is possible that ERT may improve LVEF in a subset of patients with prior systolic dysfunction, while not being able to prevent a slow long-term deterioration.

In our study, all subjects had a sinus rhythm and none was on pacemakers. However, our results support the conclusions of previous authors that noticed that cardiac conduction abnormalities may increase strikingly in older adult MPS patients (42). It is also important to notice that, while in this study no MPS III patient was included, a high prevalence of first-degree AV block was described in that MPS type (43). Although most MPS patients will not have a clinically significant conduction abnormality, resting electrocardiogram and 24 h Holter monitoring, when indicated, should be an integral part of the care of MPS patients, considering that the early recognition may have a high impact on morbidity and mortality (42).

This work has some limitations. The measurements were performed by more than one echocardiographer and the time frame of the study involved a period before the establishment of new guidelines for echocardiographic measurements in our center when newer quantification methods for LA size, ejection fraction, and LVM were included in the routine echocardiogram protocol. Nevertheless, by including data from a wide period of time, we were able to estimate the prevalence of cardiovascular abnormalities in a large sample of MPS patients as well as the long-term effectiveness of ERT.

In summary, we identified a high proportion of MPS patients with cardiac abnormalities, which ranged from isolated mitral or aortic thickening to a more severe clinical picture including moderate to severe valvular insufficiency or stenosis, left ventricular hypertrophy, pulmonary hypertension, and, rarely, systolic dysfunction. We also confirmed the impact of long-term ERT on left ventricular hypertrophy in the mucopolysaccharidoses and its limitations in reversing other cardiovascular manifestations, such as valvular involvement.

## Data Availability Statement

The raw data supporting the conclusions of this article will be made available by the authors, without undue reservation.

## Ethics Statement

The studies involving human participants were reviewed and approved by Grupo de Pesquisa e Pós-Graduação, Hospital de Clínicas de Porto Alegre, Porto Alegre, Brazil. Written informed consent from the participants' legal guardian/next of kin was not required to participate in this study in accordance with the national legislation and the institutional requirements.

## Author Contributions

FP, RG, and GB designed the study. HS and FP obtained the clinical data from chart review. AS and SB reviewed the echocardiographic and electrocardiographic data and analysis. CS reviewed the clinical data. FP drafted the manuscript. All authors read and approved the final manuscript.

## Funding

FP conducted this work during scholarship financed by CNPq—Brazil. RG and GB were recipients of CNPq—Brazil research scholarships. The work received support from The Isaac Foundation and from FIPE-HCPA (17–0013).

## Conflict of Interest

The authors declare that the research was conducted in the absence of any commercial or financial relationships that could be construed as a potential conflict of interest.

## Publisher's Note

All claims expressed in this article are solely those of the authors and do not necessarily represent those of their affiliated organizations, or those of the publisher, the editors and the reviewers. Any product that may be evaluated in this article, or claim that may be made by its manufacturer, is not guaranteed or endorsed by the publisher.

## References

[1] Kubaski F De Oliveira Poswar F Michelin-Tirelli K Burin MG Rojas-Málaga D Brusius-Facchin AC. Diagnosis of mucopolysaccharidoses. Diagnostics. (2020) 10:172. 10.3390/diagnostics10030172 32235807 PMC7151013

[2] Stapleton M Arunkumar N Kubaski F Mason RW Tadao O Tomatsu S. Clinical presentation and diagnosis of mucopolysaccharidoses. Mol Genet Metab. (2018) 125:4–17. 10.1016/j.ymgme.2018.01.003 30057281

[3] Braunlin EA Harmatz PR Scarpa M Furlanetto B Kampmann C Loehr JP. Cardiac disease in patients with mucopolysaccharidosis: presentation, diagnosis and management. J Inherit Metab Dis. (2011) 34:1183–97. 10.1007/s10545-011-9359-8 21744090 PMC3228957

[4] Baldo G Tavares AMV Gonzalez E Poletto E Mayer FQ Matte U da S. Progressive heart disease in mucopolysaccharidosis type I mice may be mediated by increased cathepsin B activity. Cardiovasc Pathol. (2017) 27:45–50. 10.1016/j.carpath.2017.01.001 28104572

[5] Parini R Deodato F. Intravenous enzyme replacement therapy in mucopolysaccharidoses: clinical effectiveness and limitations. Int J Mol Sci. (2020) 21:2975. 10.3390/ijms21082975 32340185 PMC7215308

[6] Poswar F de O de Souza CFM Giugliani R Baldo G. Aortic root dilatation in patients with mucopolysaccharidoses and the impact of enzyme replacement therapy. Heart Vessels. (2019) 34:290–5. 10.1007/s00380-018-1242-1 30136169

[7] Quartel A Harmatz PR Lampe C Guffon N Ketteridge D Leão-Teles E. Long-term galsulfase treatment associated with improved survival of patients with mucopolysaccharidosis vi (maroteaux-lamy syndrome). J Inborn Errors Metab Screen. (2018) 6:232640981875580. 10.1177/2326409818755800

[8] Haycock GB Schwartz GJ Wisotsky DH. Geometric method for measuring body surface area: a height-weight formula validated in infants, children, and adults. J Pediatr. (1978) 93:62–66. 10.1016/S0022-3476(78)80601-5 650346

[9] Devereux RB Alonso DR Lutas EM Gottlieb GJ Campo E Sachs I. Echocardiographic assessment of left ventricular hypertrophy: comparison to necropsy findings. Am J Cardiol. (1986) 57:450–8. 10.1016/0002-9149(86)90771-X 2936235

[10] Lopez L Colan S Stylianou M Granger S Trachtenberg F Frommelt P. Relationship of echocardiographic Z scores adjusted for body surface area to age, sex, race, and ethnicity. Circ Cardiovasc Imaging. (2017) 10:e006979. 10.1161/CIRCIMAGING.117.006979 29138232 PMC5812349

[11] Sluysmans T Colan SD. Structural measurements and adjustments for growth. In: Lai WW Mertens L Cohen M Geva T editors. Echocardiography in Pediatric and Congenital Heart Disease. Oxford: John Wiley & Sons, Ltd, 61–72.

[12] Boston Children's Hospital. BCH Z-Score Calculator - Home. Available online at: http://zscore.chboston.org/ (accessed May 9, 2020).

[13] Kampmann C Lampe C Whybra-Trümpler C Wiethoff CM Mengel E Arash L. Mucopolysaccharidosis VI: cardiac involvement and the impact of enzyme replacement therapy. J Inherit Metab Dis. (2014) 37:269–276. 10.1007/s10545-013-9649-4 24062198

[14] Teichholz LE Kreulen T Herman M V. Gorlin R. Problems in echocardiographic volume determinations: echocardiographic-angiographic correlations in the presence or absence of asynergy. Am J Cardiol. (1976) 37:7–11. 10.1016/0002-9149(76)90491-4 1244736

[15] Bazett HC. An analysis of the time relations of electrocardiograms. Heart. (1920) 7:353–70.

[16] Poswar FO Martins GR Giugliani R Baldo G. Aortic root dilatation in patients with mucopolysaccharidoses and the effect of enzyme replacement therapy. Mol Genet Metab. (2017) 120:S110. 10.1016/j.ymgme.2016.11.280 30136169

[17] Leal GN De Paula AC Leone C Kim CA. Echocardiographic study of paediatric patients with mucopolysaccharidosis. Cardiol Young. (2010) 20:254–261. 10.1017/S104795110999062X 20416133

[18] Chen MR Lin SP Hwang HK Yu CH. Cardiovascular changes in mucopolysaccharidoses in Taiwan. Acta Cardiol. (2005) 60:51–3. 10.2143/AC.60.1.2005049 15779852

[19] Kampmann C Abu-Tair T Gökce S Lampe C Reinke J Mengel E. Heart and cardiovascular involvement in patients with mucopolysaccharidosis type IVA (Morquio-A Syndrome). PLoS ONE. (2016) 11:e0162612. 10.1371/journal.pone.0162612 27610627 PMC5017658

[20] Hendriksz CJ Giugliani R Harmatz P Lampe C Martins AM Pastores GM. Design, baseline characteristics, and early findings of the MPS VI (mucopolysaccharidosis VI) Clinical Surveillance Program (CSP). J Inherit Metab Dis. (2013) 36:373–84. 10.1007/s10545-011-9410-9 22127392

[21] Lampe C Bosserhoff AK Burton BK Giugliani R de Souza CF Bittar C. Long-term experience with enzyme replacement therapy (ERT) in MPS II patients with a severe phenotype: an international case series. J Inherit Metab Dis. (2014) 37:823–9. 10.1007/s10545-014-9686-7 24596019 PMC4158409

[22] Lin HY Chuang CK Wang CH Chien YH Wang YM Tsai FJ. Long-term galsulfase enzyme replacement therapy in Taiwanese mucopolysaccharidosis VI patients: a case series. Mol Genet Metab Rep. (2016) 7:63–9. 10.1016/j.ymgmr.2016.04.003 27134829 PMC4834679

[23] Braunlin EA Berry JM Whitley CB. Cardiac findings after enzyme replacement therapy for mucopolysaccharidosis type I. Am J Cardiol. (2006) 98:416–8. 10.1016/j.amjcard.2006.02.047 16860035

[24] Braunlin E Rosenfeld H Kampmann C Johnson J Beck M Giugliani R. Enzyme replacement therapy for mucopolysaccharidosis VI: long-term cardiac effects of galsulfase (Naglazyme®) therapy. J Inherit Metab Dis. (2013) 36:385–94. 10.1007/s10545-012-9481-2 22669363 PMC3590402

[25] Lin HY Chuang CK Chen MR Lin SM Hung CL Chang CY. Cardiac structure and function and effects of enzyme replacement therapy in patients with mucopolysaccharidoses I, II, IVA and VI. Mol Genet Metab. (2016) 117:431–7. 10.1016/j.ymgme.2016.02.003 26899310

[26] Gabrielli O Clarke LA Ficcadenti A Santoro L Zampini L Volpi N Coppa G V. 12year follow up of enzyme-replacement therapy in two siblings with attenuated mucopolysaccharidosis I: the important role of early treatment. BMC Med Genet. (2016) 17:19. 10.1186/s12881-016-0284-4 26965916 PMC4785727

[27] Baldo G Mayer FQ Martinelli BZ de Carvalho TG Meyer FS de Oliveira PG. Enzyme replacement therapy started at birth improves outcome in difficult-to-treat organs in mucopolysaccharidosis I mice. Mol Genet Metab. (2013) 109:33–40. 10.1016/j.ymgme.2013.03.005 23562162

[28] Boffi L Russo P Limongelli G. Early diagnosis and management of cardiac manifestations in mucopolysaccharidoses: a practical guide for paediatric and adult cardiologists. Ital J Pediatr. (2018) 44:122. 10.1186/s13052-018-0560-3 30442163 PMC6238246

[29] Monda E Rubino M Lioncino M Di Fraia F Pacileo R Verrillo F. Hypertrophic cardiomyopathy in children: pathophysiology, diagnosis, and treatment of non-sarcomeric causes. Front Pediatr. (2021) 9:632293. 10.3389/fped.2021.632293 33718303 PMC7947260

[30] Yano S Li C Pavlova Z. The transforming growth factor-Beta signaling pathway involvement in cardiovascular lesions in mucopolysaccharidosis-I. JIMD Rep. (2013) 7:55–8. 10.1007/8904_2012_141 23430495 PMC3575049

[31] Braunlin E Wang R. Cardiac issues in adults with the mucopolysaccharidoses: current knowledge and emerging needs. Heart Br Card Soc. (2016) 102:1257–62. 10.1136/heartjnl-2015-309258 27102649

[32] Poorthuis BJHM Romme AE Willemsen R Wagemaker G. Bone marrow transplantation has a significant effect on enzyme levels and storage of glycosaminoglycans in tissues and in isolated hepatocytes of mucopolysaccharidosis type VII mice. Pediatr Res. (1994) 36:187–93. 10.1203/00006450-199408000-00009 7970933

[33] Braunlin E Mackey-Bojack S Panoskaltsis-Mortari A Berry JM McElmurry RT Riddle M. Cardiac functional and histopathologic findings in humans and mice with mucopolysaccharidosis type I: implications for assessment of therapeutic interventions in hurler syndrome. Pediatr Res. (2006) 59:27–32. 10.1203/01.pdr.0000190579.24054.39 16326988

[34] Waggoner AD Adyanthaya AV Quinones MA Alexander JK. Left atrial enlargement. Echocardiographic assessment of electrocardiographic criteria. Circulation. (1976) 54:553–7. 10.1161/01.CIR.54.4.553 134852

[35] Lin HY Chen MR Lin SM Hung CL Niu DM Chuang CK. Cardiac features and effects of enzyme replacement therapy in Taiwanese patients with Mucopolysaccharidosis IVA Dr. Segolene Ayme. Orphanet J Rare Dis. (2018) 13:148. 10.1186/s13023-018-0883-6 30157891 PMC6114849

[36] Bolourchi M Renella P Wang RY. Aortic root dilatation in mucopolysaccharidosis I-VII. Int J Mol Sci. (2016) 17:2004. 10.3390/ijms17122004 27916847 PMC5187804

[37] Yabek SM Isabel-Jones J Bhatt DR Nakazawa M Marks RA Jarmakani JM. Echocardiographic determination of left atrial volumes in children with congenital heart disease. Circulation. (1976) 53:268–72. 10.1161/01.CIR.53.2.268 1245035

[38] John Â Fagondes S Schwartz I Azevedo AC Barrios P Dalcin P. Sleep abnormalities in untreated patients with mucopolysaccharidosis type VI. Am J Med Genet A. (2011) 155:1546–51. 10.1002/ajmg.a.33902 21638759

[39] Rossi A Dini FL Agricola E Faggiano P Benfari G Temporelli PL. Left atrial dilatation in systolic heart failure: a marker of poor prognosis, not just a buffer between the left ventricle and pulmonary circulation. J Echocardiogr. (2018) 16:155–61. 10.1007/s12574-018-0373-9 29476388

[40] Okuyama T Tanaka A Suzuki Y Ida H Tanaka T Cox GF. Japan Elaprase® treatment (JET) study: idursulfase enzyme replacement therapy in adult patients with attenuated Hunter syndrome (Mucopolysaccharidosis II, MPS II). Mol Genet Metab. (2010) 99:18–25. 10.1016/j.ymgme.2009.08.006 19773189

[41] Parini R Rigoldi M Tedesco L Boffi L Brambilla A Bertoletti S. Enzymatic replacement therapy for Hunter disease: up to 9 years experience with 17 patients. Mol Genet Metab Rep. (2015) 3:65–74. 10.1016/j.ymgmr.2015.03.011 26937399 PMC4750582

[42] Ayuna A Stepien KM Hendriksz CJ Balerdi M Garg A Woolfson P. Cardiac rhythm abnormalities - an underestimated cardiovascular risk in adult patients with Mucopolysaccharidoses. Mol Genet Metab. (2020) 130:133–9. 10.1016/j.ymgme.2020.03.005 32241717

[43] Nijmeijer SCM de Bruin-Bon RHACM Wijburg FA Kuipers IM. Cardiac disease in mucopolysaccharidosis type III. J Inherit Metab Dis. (2019) 42:276–85. 10.1002/jimd.12015 30671988

