# Supplementary figures and images for: Progression of Cardiovascular Manifestations in Adults and Children With Mucopolysaccharidoses With and Without Enzyme Replacement Therapy
Source: Front Cardiovasc Med. 2022 Jan 12;8:801147. doi: 10.3389/fcvm.2021.801147 (PMC8790121; doi:10.3389/fcvm.2021.801147)

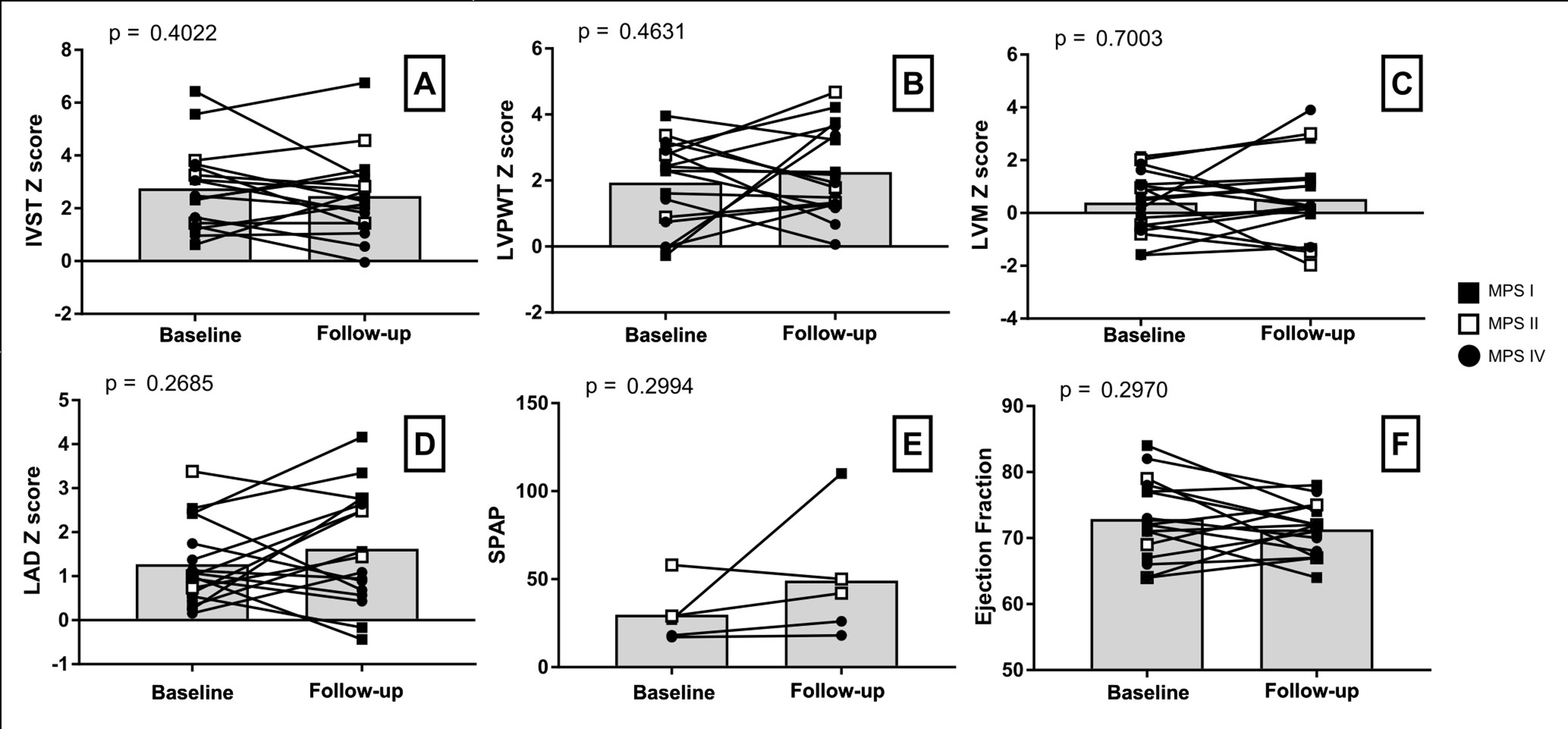

Supplement: Supplementary file 1 [file Image_1.JPG]
