# Supplementary material for: Progression of Cardiovascular Manifestations in Adults and Children With Mucopolysaccharidoses With and Without Enzyme Replacement Therapy
Source: Front Cardiovasc Med. 2022 Jan 12;8:801147. doi: 10.3389/fcvm.2021.801147 (PMC8790121; doi:10.3389/fcvm.2021.801147)
Supplement: Supplementary file 2 [file Data_Sheet_1.docx]

**SUPPLEMENTARY FIGURE LEGEND**

**Figure S1.** Measurements of echocardiographic parameters among MPS types I, II and IV before and after at least 18 months of follow-up for single individuals that remained untreated (A-F), including the Z scores of interventricular septum thickness (A), left ventricle posterior wall thickness (B), left ventricle mass (C), left atrium diameter (D), systolic pulmonary artery pressure (E) and ejection fraction (F). Statistical analysis with paired t test.
